# Role of NOD-Like Receptors in a Miniature Pig Model of Diabetic Renal Injuries

**DOI:** 10.1155/2022/5515305

**Published:** 2022-03-30

**Authors:** Yi Ren, Shaoyuan Cui, Quan Hong, Wanjun Shen, Qinggang Li, Lingling Wu, Bo Fu, Xu Wang, Qian Ma, Jiaona Liu, Xueyuan Bai, Xiangmei Chen

**Affiliations:** Medical School of Chinese PLA, Department of Nephrology, The First Medical Centre, Chinese PLA General Hospital, Chinese PLA Institute of Nephrology, State Key Laboratory of Kidney Diseases, National Clinical Research Center for Kidney Diseases, Beijing Key Laboratory of Kidney Diseases, Beijing 100853, China

## Abstract

Activation of NOD-like receptor (NLR) signaling pathway can promote downstream cytokine and proinflammatory cytokines release, and inflammation induced by excess nutrients leads to renal metabolic injury. How the NLRs influence metabolic progress and then lead to the renal injury remains poorly investigated. Compared with rodents, minipigs are more similar to humans and are more ideal animal models for human disease research. In this study, we established a diabetic minipig model through a high-sugar and high-fat diet combined with streptozotocin (STZ) injection. Blood biological markers and renal pathological markers, expression of NLRP subfamily members (NLRP1 and NLRP3) and their downstream cytokines (precursors of IL-1*β* and IL-18 and mature forms of IL-1*β* and IL-18), expression of NLRC subfamily members (NLRC1, NLRC2, and NLRC5) and their downstream nuclear factor-*κ*B (NF-*κ*B) signaling pathway molecules (IKK*β*, I*κ*B*α*, and NF-*κ*B p65), and inflammatory cytokines (TNF-*α* and interleukin-6 (IL-6)) were systematically evaluated. The expression of NLRP3 and its downstream cytokine signaling molecules, the precursors of IL-1*β* and IL-18, and the mature forms of IL-1*β* and IL-18 was significantly upregulated. The expression levels of NLRC1, NLRC2, and NLRC5 and activation of the downstream NF-*κ*B pathway molecules phospho-IKK*β*, phospho-I*κ*B*α*, NF-*κ*B p65, and phospho-NF-*κ*B p65 were significantly increased. The TNF-*α* and IL-6 levels were significantly increased in diabetic pig kidneys. The TGF-*β*/Smad signaling molecules, TGF-*β* and P-SMAD2/3, were also increased. These results suggested that the metabolic inflammation activated by NLRs might play an important role in diabetic renal injuries.

## 1. Introduction

Diabetic nephropathy (DN) is the main cause of end-stage kidney disease (ESRD). Approximately 43.8% are ESRD patients whose first diagnosis was diabetes [1]. DN is a disease that could cause progressive renal fibrosis or sclerosis, leading to chronic renal failure [2]. Although there have been many related studies in recent years, the specific pathogenesis of diabetic nephropathy is still unclear.

Currently, both innate immune factors and adaptive immune factors are believed to be related to the pathogenesis of type 2 DM [3]. The innate immune system includes several classes of pattern recognition receptors (PPRs), including the membrane-bound Toll-like receptors (TLRs), which function through pathogen-associated molecular patterns (PAMPs), and nucleotide-binding oligomerization domain NOD-like receptors (NLRs), which function through danger-associated molecular patterns (DAMPs), which regulate the expression of proinflammatory cytokines and chemokines in the extracellular space [4, 5]. NLRs can be divided into two subfamilies, NLRP and NLRC [6].

The NLRP subfamily includes 14 members. The NLRP protein oligomerizes through homomolecular interactions and recruits the PYD-CARD adaptor protein, ASC, and the protease caspase-1 to form a protein complex called the inflammasome [7, 8]. The inflammasome can activate caspase-1 by proteolytic cleavage of procaspase-1. Activated caspase-1 converts the precursor forms of the cytokines IL-1*β* and IL-18 into the mature forms and secretes these cytokines into the extracellular matrix [9, 10]. The NLRC subfamily recruits receptors by its CARD to interact with protein kinase receptor-interacting protein 2 (RIP2) receptors by recognizing lipopolysaccharides and peptidoglycans in bacterial cell walls. This molecule activates mitogen-activated protein kinases (MAPKs) and the nuclear transcription factor NF-*κ*B [11]. Studies have confirmed that members of the NLRP subfamily aggravate the inflammatory response of necrotic cells in renal ischemia-reperfusion injury [12], and the absence of NLRC subfamily members can significantly improve ischemia-reperfusion renal injury by reducing renal cell apoptosis [13]. However, the role that these NLR family members play in the development of diabetic kidney damage remains unclear.

Rodents are the classic model animal for diabetes researching [14]. However, due to the obvious differences in body size, behavior, lifespan, and living conditions between humans and mice, there are many shortcomings in the disease models of rodents. Miniature pigs (minipigs) are considered to be a better choice than rodents due to their high similarity to humans (gastrointestinal system and immune system) in anatomy (body size, cardiovascular system, skin, and urinary system) and function [15, 16]. Pig kidneys are more similar in morphology and function to human kidneys. The number of small calyxes in the collection system and the bilaterally or mirrored structure of the renal pelvis system of minipigs is similar to that in humans [17]. Therefore, pigs, especially minipigs, are ideal model for studying human kidney diseases.

In this study, we established a diabetic kidney injury model by using low-dose streptozotocin (STZ) injection and high-sugar and high-fat feeding in Bama minipigs. We aimed to systematically investigate the changes in the two subfamilies of NLRs and downstream inflammation-associated signaling pathway molecules in the kidney tissues in minipig diabetic models.

## 2. Materials and Methods

### 2.1. Animals and Environmental Conditions

A total of 14 male Chinese Bama experimental minipigs aged 4 months were obtained from the State Key Laboratory for the Institute of Zoology, Chinese Academy of Sciences. The housing conditions were as follows: room temperature of 18–22°C and humidity of 30% to 70% with artificial lighting from 8 : 00 to 20 : 00 hours. All animals involved in the experiments were approved by the Institutional Animal Care and Use Committee at the Chinese PLA General Hospital.

### 2.2. Experimental Groups and Intervention

Minipigs were randomly divided into two groups: the DM (*n* = 8) group, which was fed a high-sugar and high-fat diet comprising 53% basal diet, 37% sucrose, and 10% lard and the control group (CON; *n* = 6), which was fed a basal diet. The amount of daily feeding was 3% of the minipigs' weight, twice a day. Water was available ad libitum. The DM group was injected with streptozotocin (STZ) in the first month. We mixed 28 ml of citric acid with 22 ml of trisodium citrate and diluted 100 ml of the buffer to pH 4.5 with distilled water. According to the animal body mass, a certain amount was measured and prepared in advance. The prepared buffer had 25 g/l STZ and was filtered. Fasting minipigs ate overnight before they were injected with STZ. The injection was divided into 2 doses. The 50 mg/kg dose of STZ was injected intravenously into each pig for the first time, within time 1-5 min. The second injection was performed two weeks later with the same dose. The control group pigs were injected with the same dosage of citrate-sodium buffer.

### 2.3. Serum Biochemical Analysis

The blood samples were collected monthly from the veins of the pigs to measure fasting glucose (GLU) during feeding. The serum samples were collected by centrifugation at 3,000 rpm for 10 min and stored in a −80°C freezer before analysis. Serum biochemical parameters were analyzed at the Central Laboratory by an autoanalyzer (Cobas8000, Roche, Germany). Blood glucose was detected by o-toluidine method; insulin was detected by enzyme-linked immunosorbent assay. Blood urea nitrogen was detected by diacetyl-oxime method, and creatinine was detected by alkaline picric acid colorimetric method. TG, CHOL, HDL-C, and LDL-C were determined by GPO-PAP method.

### 2.4. Pathological Analysis of Renal Tissues

Minipigs were anesthetized by continuous inhalation of hydrated isoflurane using a face mask, and the kidney tissues were excised. The tissues were fixed in 4% paraformaldehyde, embedded in paraffin, and sectioned at 3-5 mm. Periodic acid-Schiff (PAS) staining was used to evaluate the pathological changes in the renal structures of the minipigs.

### 2.5. Western Blot

Frozen kidney tissues were lysed with radioimmunoprecipitation assay (RIPA) lysis buffer (55 mM Tris-Cl (pH 7.6), 1% NP-40, leupeptin 1 *μ*g/ml, 0.1% SDS, 0.5% deoxycholic acid, and phenylmethyl-sulfonyl fluoride), centrifuged at 12,000 g for 30 min at 4°C to collect cellular proteins in the supernatants. The protein concentration was determined by a BCA protein assay kit (Thermo Fisher Scientific). Approximately 40 *μ*g of protein from each sample was separated by 8%-15% SDS-PAGE. The samples were transferred from the SDS-PAGE gels to membranes. The membranes were blocked and incubated in antibodies against NLRP3 (1 : 1000, Abcam, ab264468), procaspase-1 (1 : 1000,CST,3866), NLRP1 (1 : 1000, Abcam, ab36852), caspase-1 (1 : 1000, CST, 4199), pro-IL-1*β* (1 : 1000, CST, 12703), IL-1*β* (1 : 1000, CST, 83186), pro-IL-18 (1 : 1000, Abcam, ab241528), IL-18 (1 : 1000, CST, 54943), NLRC1 (1 : 1000, CST, 3545), NLRC2 (1 : 500, Abcam, ab31488), NLRC5 (1 : 1000, Santa Cruz Biotechnology, Sc-515668), phospho-IKK*β* (1 : 1000, Abcam, ab59195), phospho-I*κ*B*α* (1 : 1000, CST, 9246), NF-*κ*Bp65(1 : 1000, Abcam, ab31481), phospho-NF-Bp65 (1 : 1000, CST, 3033), TNF-*α* (1 : 1000, Abcam, ab183218), IL-6 (1 : 1000, CST, 12153), TGF-*β* (1 : 1000, Abcam, ab215715), P-SMAD2/3 (1 : 1000, Abcam, ab272332), and *β*-actin (1 : 1000, CST, 4967) overnight at 4°C. The blots were subsequently incubated with horseradish peroxidase-conjugated anti-rabbit or anti-mouse immunoglobulin at dilutions of 1 : 1000 to 1 : 4000. Immunoreactive bands were visualized via enhanced chemiluminescence, and densitometry was performed using Quantity One software (Bio-Rad Laboratories). ImageJ was used for blot analysis.

### 2.6. Statistical Analysis

Statistical analysis was performed using the SPSS 21.0 statistical software package, and all statistical data are presented as *x* ± SD. Student's *t* test was carried out for comparisons between two independent groups. A value of *P* < 0.05 was considered to indicate statistical significance.

## 3. Results

### 3.1. Blood and Urinary Biochemical Changes in the Minipig Model of Diabetes

After 8 months of STZ injection and feeding with high-sugar and high-fat, the blood glucose (GLU) and insulin (INS) levels of the DM group minipigs were significantly increased (*P* < 0.05) compared with those of the normal control (CON) group. The levels of cholesterol (CHOL) and low-density lipoprotein cholesterol (LDL-C) were also substantially increased (*P* < 0.05) in the DM group minipigs. However, there were no significant changes in the levels of urea nitrogen and creatinine between the DM and CON groups (*P* > 0.05) (see Table 1).

### 3.2. Pathological Changes in the Kidneys of the Diabetic Minipig Models

**Figure 1:**
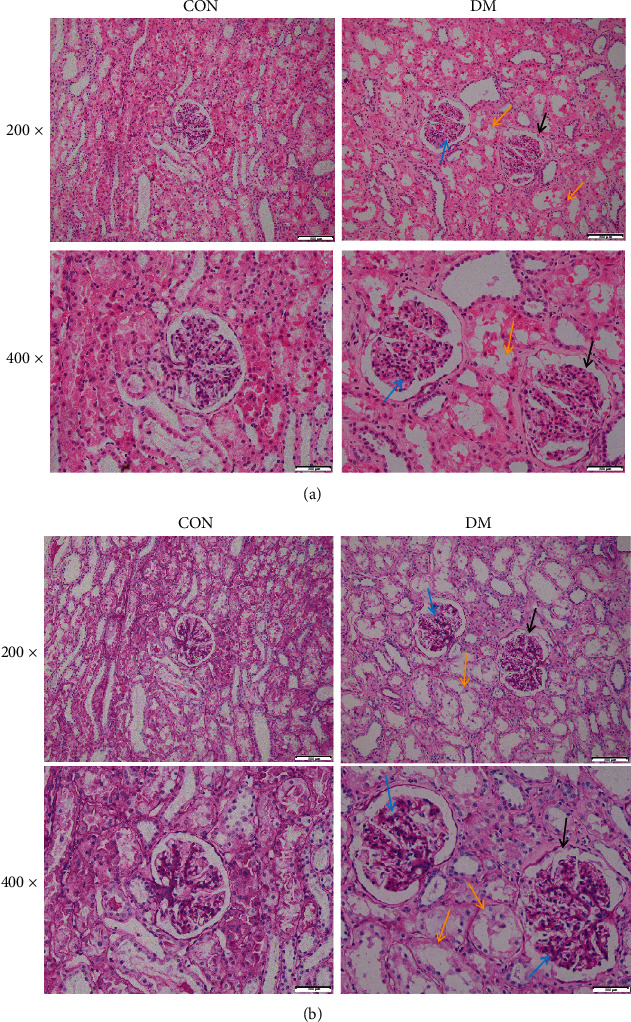
Optical microscope results of the kidneys of minipigs (magnification: 200× and 400×). (a) H&E staining results of the kidneys of minipigs. (b) PAS staining results of the kidneys of minipigs (black arrows: glomerular hypertrophy; blue arrows: increased mesangial matrix; yellow arrows: increased renal tubule diameter).

**Figure 2:**
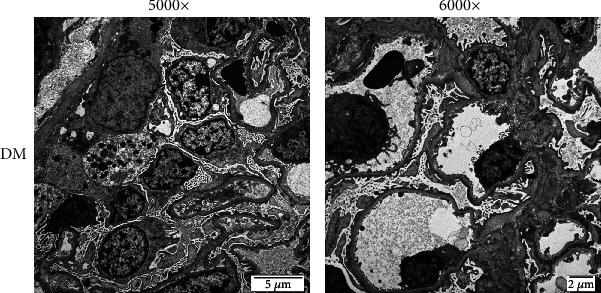
Electron microscope results of the kidneys of minipigs (magnification: 5000× and 6000×).

The kidney tissues of the animals were observed pathologically and stained with H&E and PAS staining. The kidneys of the minipigs in the DM group showed obvious pathologic changes, including glomerular hypertrophy, increased mesangial matrix, and an enlarged renal tubule diameter, whereas there were no obvious histological alterations in the kidneys of the CON group (see Figure 1). The fusion of foot processes on epithelial cell can be observed with an electron microscope (see Figure 2).

### 3.3. Expression of the NLRP Subfamily and Downstream Molecules Increased in the Kidneys of the Diabetic Minipigs

The NLRP subfamily can interact with procaspase-1 to assemble into high-molecular weight inflammasomes, and then, the inflammasomes can activate procaspase-1, which is converted to caspase-1 through enzymatic cleavage. Activated caspase-1 can promote the maturation and secretion of pro-IL-1*β* and pro-IL-18, which play an important role in the endogenous immune response. We used the Western blot analysis to assess the expression of NLRP1 and NLRP3 which are representative members of the NLRP subfamily and the expression of procaspase-1 and caspase-1, precursors of IL-1*β* and IL-18, and the mature forms of IL-1*β* and IL-18.

**Figure 3:**
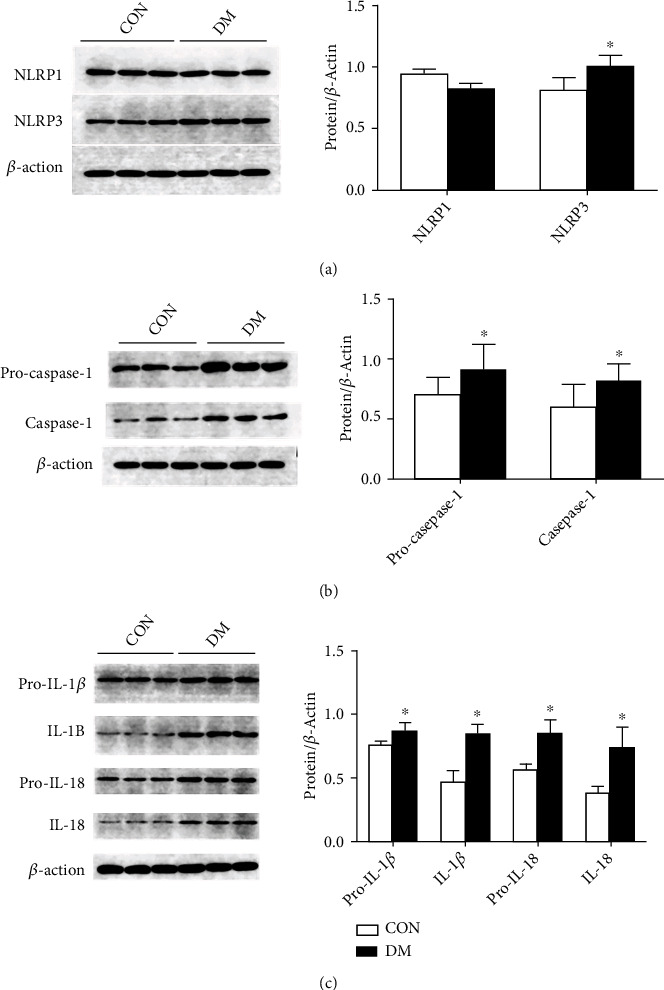
Expression of the NLRP subfamily and downstream molecules was analyzed by Western blots in kidney tissues from diabetic minipigs. (a) Detection of NLRP subfamily members' expression by Western blots and semiquantitative analysis of the expression levels of NLRP subfamily members. (b) Detection of procaspase-1 and caspase-1 expression by Western blots and semiquantitative analysis of the expression levels of procaspase-1 and caspase-1. (c) Detection of downstream cytokine expression by Western blots and semiquantitative analysis of the expression levels of downstream cytokines. Protein expression data are presented as the means ± SD. ^∗^*P* < 0.05 versus CON.

The results showed that the levels of NLRP3 in the kidneys of the diabetes group were higher than those in the normal group (*P* < 0.05), but the level of NLRP1 did not change significantly compared with that of the normal group (*P* > 0.05) (Figure 3(a)). The results also showed that the levels of procaspase-1, caspase-1, pro-IL-1*β*, pro-IL-18, IL-1*β*, and IL-18 in the kidneys of the diabetic group were higher than those in the normal control group (*P* < 0.05), indicating that the NLRP inflammasome increases during diabetic kidney damage (Figure 3(b)), which activates the release of downstream proinflammatory mediators (Figure 3(c)).

### 3.4. Expression of the NLRC Subfamily and Downstream Signaling Pathway Molecules Was Upregulated in the Kidneys of the Diabetic Minipigs

The NLRC subfamily can activate the NF-*κ*B signaling pathway and regulate the release of inflammatory factors such as TNF-*α* and IL-6 in monocytes. We used Western blotting to detect the changes in the expression of NLRC1, NLRC2, and NLRC5, which are representative members of the NLRC subfamily. Then, we further assessed the expression or activation statuses of the NF-*κ*B signaling pathway molecules IKK*β*, I*κ*B, and NF-*κ*B and the expression changes in the inflammatory factors TNF-*α* and IL-6 downstream of NLRC.

**Figure 4:**
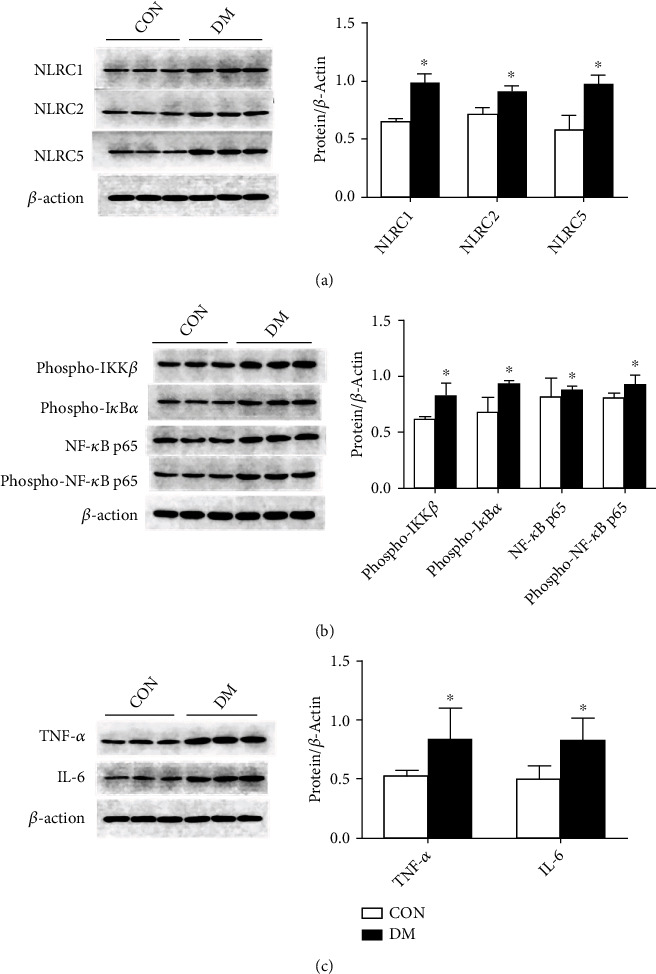
Expression of the NLRC subfamily and the downstream NF-*κ*B signaling pathway was analyzed by Western blots in kidney tissues from diabetic minipigs. (a) Detection of NLRC subfamily members' expression by Western blots and semiquantitative analysis of the expression levels of the NLRC subfamily members. (b) Detection of the NF-*κ*B signaling pathway by Western blots and semiquantitative analysis of the expression levels of the NF-*κ*B signaling pathway. (c) Detection of inflammatory factor (TNF-*α* and IL-6) expression by Western blots and semiquantitative analysis of the expression levels of inflammatory factors (TNF-*α* and IL-6). Protein expression data are presented as the means ± SD. ^∗^*P* < 0.05 versus CON.

The expression levels of NLRC1, NLRC2, and NLRC5 in the NLRC subfamily were higher than those in the normal control group (*P* < 0.05) (Figure 4(a)), suggesting that the levels of NLRC subfamily members are increased in minipigs with diabetic renal injuries. The results also showed that in the diabetic kidneys, the levels of the NF-*κ*B signaling pathway molecules phospho-IKK*β* (activated IKK*β*), phospho-I*κ*B*α* (activated I*κ*B*α*), NF-*κ*B p65, and phospho-NF-*κ*B p65 (activated NF-*κ*B p65) were significantly higher than those in the CON kidneys (Figure 4(b)), indicating that the NF-*κ*B signaling pathway was activated during the development of diabetic kidney lesions. Moreover, the levels of TNF-*α* and IL-6 in the kidneys of the diabetic group were higher than those in the normal control group (*P* < 0.05), which indicated that the release of the inflammatory factors TNF-*α* and IL-6 increased when the diabetic kidney was damaged (Figure 4(c)).

### 3.5. Expression of the TGF-*β*/Smad Signaling Pathway Molecules Was Upregulated in the Kidneys of the Diabetic Minipigs

TGF-*β* (transforming growth factor *β*) is a multifunctional growth factor. As an effector molecule, TGF-*β* has been widely studied as the main mediator in diabetic nephropathy. Smad protein is a downstream mediator molecule of intracellular signal transduction. We used Western blotting to detect the expression of TGF-*β*/Smad signaling pathway molecules.

**Figure 5:**
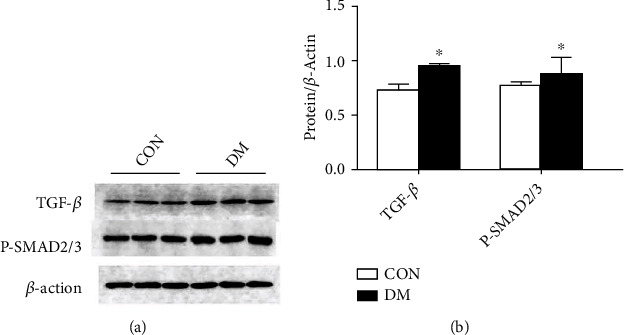
Expression of TGF-*β* and P-SMAD2/3 was analyzed by Western blots in kidney tissues from diabetic minipigs. (a) Detection of TGF-*β* and P-SMAD2/3 expression by Western blots. (b) Semiquantitative analysis of the expression levels of TGF-*β* and P-SMAD2/3. Protein expression data are presented as the means ± SD. ^∗^*P* < 0.05 versus CON.

The expression levels of TGF-*β* and P-SMAD2/3 were higher than those in the normal control group (*P* < 0.05) (Figure 5), indicating that the TGF-*β*/Smad signaling pathway was activated during the development of diabetic kidney lesions.

## 4. Discussion

Currently, the number of diabetic patients worldwide is increasing, and the cause is still unclear. Many experimental animals have been used for diabetes research. For example, primate macaques are similar to humans and ideal for researching diabetes, but the costs are high. Rodents, such as mice and rats, are the most common used animals for research, but their organs and functions are quite different from those of humans. Currently, minipigs have been increasingly used to establish diabetic models. The main reason is that minipigs are very similar to humans in function and structure. The amino acid composition of the INS peptide chain of minipigs is very similar to that of humans, with only one amino acid difference (located at the 30th amino acid of the B chain; humans have threonine at this position, and pigs have alanine) [18]. In terms of the cardiovascular system, the morphological structure, system source, and physiological characteristics of minipigs are also similar to those of humans [19]. In terms of diet, pigs are omnivorous animals, the same as humans, and their metabolic characteristics, such as carbohydrate and lipid metabolism, are also similar to those of humans [20]. Compared with mice and other rodents, minipigs are more similar to humans in the structure of the gastrointestinal tract, subcutaneous drug management, and the morphology of pancreatic islets and have many similarities to humans in terms of metabolism and pharmacokinetics. Therefore, minipigs are ideal animals for studying diabetes and its complications.

High-sugar and high-fat feeding combined with STZ injection is a common method to induce type 2 diabetes models [21]. Small pigs fed with high-fat diet and intravenously injected with STZ resulted in a stable animal model of type 2 diabetes [22], which is characterized by moderate hyperglycemia, hyperlipidemia, hypertension, and INS resistance. This method in minipigs can be regarded as an ideal choice for establishing a diabetes model. This finding is consistent with our method of establishing disease models and the observed phenomena. We used stable strains of Bama minipigs given high-sugar and high-fat diet combined with STZ injection to establish a disease model. We observed that compared with those of the normal control group, the blood GLU level and INS level of the diabetic minipigs increased significantly (*P* < 0.05). The PAS staining results showed that the diabetic model group had pathological changes with obvious glomerular hypertrophy and an enlarged renal tubule diameter as the main manifestations, which were consistent with the pathological manifestations of type 2-related diabetes kidney damage, while the normal control group showed no obvious changes in structure. The above results showed that we established a stable minipig model of type 2 diabetic kidney injury.

Metabolic inflammation mainly refers to an inflammatory process caused by excess nutrients and metabolism. The molecular mechanism and signaling pathways of this process have many similarities to those of traditional inflammatory reactions, and this phenomenon is different from a slow inflammatory response [23, 24]. In recent years, studies have shown that immune receptor-mediated signaling pathways also play an important role in metabolic inflammation in diabetes [25]. The NOD-like receptors (NLRs) belong to the class of intracellular sensor molecules [26]. Some NOD-like receptors, after being stimulated and activated, form a larger protein complex called the “inflammasome” [27]. The inflammasome can convert the precursor caspase-1 to activated caspase-1, which can further cleave the precursors of inflammatory factors such as IL-1*β* and IL-18, generating the mature forms and finally releasing them to the extracellular space, which triggers an inflammatory response. The NLRP subfamily members, NLRP1 and NLRP3, are involved in the assembly of inflammasomes. Chronic inflammation may be an important pathogenic mechanism of type 2 diabetes. In addition to glucotoxicity and lipotoxicity, the NLRP3 inflammasome may play an important role in this process [28]. We detected the expression of NLRP subfamily members in the diabetic kidneys of minipigs, the inflammatory components procaspase-1 and caspase-1, and the downstream inflammatory factors pro-IL-1*β* and IL-1*β* and pro-IL-18 and IL-18. The results showed that the expression of these proteins was significantly upregulated in the diabetic group, indicating that the inflammasome was activated to release inflammatory factors during diabetic kidney injury. The body was in a metabolic inflammatory state.

The main cause of metabolic inflammation is immune abnormalities caused by excessive intake of nutrients [29]. The molecular and cellular basis of metabolic inflammation includes NF-*κ*B, the C-Jun N-terminal kinase (JNK) pathway, and endoplasmic reticulum stress. NF-*κ*B, a common factor, has been confirmed by multiple studies in the inflammatory response, and it plays a key role as a core transcription factor in a variety of regulatory pathways. Activated NF-*κ*B is involved in the relocation of macrophages and further activates the inflammatory response pathway. During injury, the inhibitor of kappaB (I*κ*B) will be separated and enter the nucleus after NF-*κ*B is stimulated. This molecule can regulate gene transcriptional activation and induce cell synthesis pathways such as TNF-*α* and IL-6 and other macromolecules after specifically binding to DNA [30]. Studies have shown that overnutrition can induce the nuclear transcription factor NF-*κ*B to promote the expression of a variety of immune-inflammatory factors, such as TNF-*α* and IL-6, and trigger the tissue to enter a state of high immune-inflammatory activation [31]. We detected the protein expression of the NF-*κ*B pathway and downstream inflammatory factors in the diabetic kidneys of minipigs. We found that the levels of phospho-I*κ*B*α*, phospho-IKK*β*, phospho-NF-*κ*B p65, NF-*κ*B p65, TNF-*α*, and IL-6 were significantly upregulated in the diabetic group compared with the normal control group, indicating that the NF-*κ*B signaling pathway was activated, and the inflammatory factors were released in the kidneys of diabetic minipigs.

Several evidences have shown that TGF-*β* is an important mediator of diabetic nephropathy. In rodent diabetes models, the levels of TGF-*β*1 mRNA and protein in the glomeruli and renal tubules are increased [32, 33]. In the TGF-*β*/Smad signaling pathway, TGF-*β* binds to the T*β*RII receptor and phosphorylates it and then forms a TGF-*β* complex by binding to T*β*RІ receptor. The complex further activates the downstream signaling proteins Smad2 and Smad3 and is finally transported to the nucleus and directly combines with DNA, regulating the regulation of transcription of target genes [34, 35]. In rodents with diabetic nephropathy, the intracellular Smad pathway is significantly activated, transducing the TGF-*β* signal [36]. We detected the protein expression of TGF-*β*/Smad pathway factors in the diabetic kidneys of miniature pigs. We found that the levels of TGF-*β* and P-SMAD2/3 were upregulated in the diabetic group compared with the normal control group, indicating that the TGF-*β*/Smad signaling pathway was activated in the kidneys of diabetic minipigs, which is consistent with the changes in rodents.

## 5. Conclusions

Taken together, we successfully established a diabetic miniature swine model and demonstrated that two NLR subfamilies, NLRPs and NLRCs, were activated and then promoted downstream inflammatory factor expression and secretion to cause metabolic inflammation in kidney tissues, ultimately leading to the occurrence and development of diabetic renal damage. This study provides a foundation for the future development of inhibitors that target NLR signaling pathway molecules and for their use in the treatment of diabetic renal injuries.

## Figures and Tables

**Table 1:** Blood biochemical results in miniature swine diabetes model.

Parameters	CON (*n* = 6)	DM (*n* = 8)
GLU(mmol/l)	5.14 ± 0.87	9.23 ± 0.76^∗^
INS (*μ*IU/ml)	4.89 ± 1.35	22.93 ± 12.9^∗^
BUN(mmol/l)	8.29 ± 1.47	4.79 ± 2.01
Cr (*μ*mol/l)	187.4 ± 20.8	118.68 ± 39.25
TG (mmol/l)	0.28 ± 0.05	0.31 ± 0.09
CHOL (mmol/l)	1.32 ± 0.15	2.21 ± 0.61^∗^
HDL-C (mmol/l)	0.39 ± 0.08	0.42 ± 0.21
LDL-C (mmol/l)	0.73 ± 0.06	1.20 ± 0.30^∗^

Values are means ± SD. CON: normal control group; DM: diabetes group. ^∗^*P* < 0.05 versus CON.

## Data Availability

The data used to support the findings of this study are included within the article.
